# Nontargeted metabonomics analysis of *Scorias spongiosa* fruiting bodies at different growth stages

**DOI:** 10.3389/fmicb.2024.1478887

**Published:** 2024-10-30

**Authors:** Xiang Nong, Shengnan Zhong, Lanying Huang, Jie Xiao, Ye Hu, Yue Xie

**Affiliations:** ^1^Forestry and Bamboo Industry Science and Technology Innovation Research Institute, Leshan Normal University, Leshan, China; ^2^Provincial Engineering and Technology Research Center for Innovative Development of Bamboo Fiber Nutrition, Leshan Normal University, Leshan, China; ^3^Bamboo Diseases and Pest Control and Resources Development Key Laboratory of Sichuan Province, Leshan, China; ^4^School of Life Science, Leshan Normal University, Leshan, China; ^5^Biotechnology and Nuclear Technology Research Institute, Sichuan Academy of Agricultural Sciences, Chengdu, China; ^6^Department of Parasitology, College of Veterinary Medicine, Sichuan Agricultural University, Chengdu, China

**Keywords:** *Scorias spongiosa*, nontargeted metabonomic analysis, sugar metabolism, bioactive substances, different growth stages

## Abstract

**Introduction:**

*Scorias spongiosa* is an edible fungus.

**Methods:**

In this study, a nontargeted metabonomic analysis was conducted on the fruiting bodies of this fungus at five growth stages, and the differences in metabolites and the related metabolic pathways during growth and development were analysed.

**Results:**

This study revealed that the five growth stages of *S. spongiosa* fruiting bodies were associated with 15 pathways. These 15 metabolic pathways are speculated to play important roles in the growth of *S. spongiosa* fruiting bodies. Eleven bioactive substances were identified among the differentially expressed compounds. The content of six bioactive substances was highest at the S1 growth stage among all the growth stages. The metabolites related to sugar metabolism were enriched in three main pathways: pentose and gluconate interconversions, the pentose phosphate pathway, and the citrate cycle (TCA cycle).

**Discussion:**

These results suggested that the S1 growth stage can be selected as the harvest period of *S. spongiosa* in fruiting bodies to retain most of the bioactive substances. Pentose and gluconate interconversions, the pentose phosphate pathway, and the TCA cycle are related to changes in polysaccharide content during the growth of *S. spongiosa* fruiting bodies.

## Graphical abstract

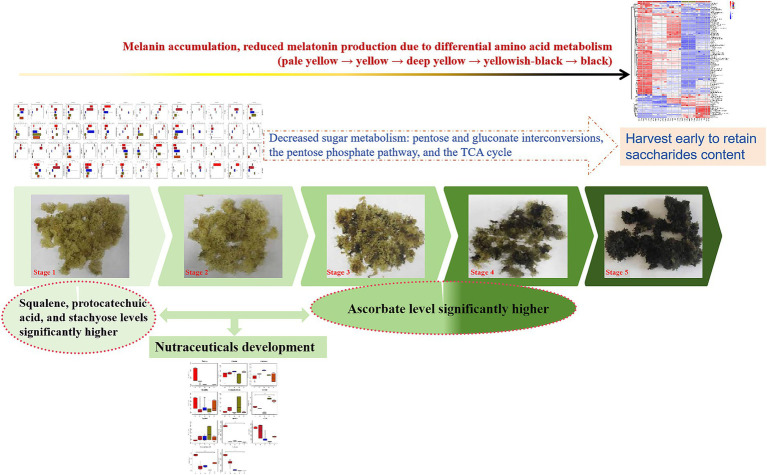

## 1 Introduction

*Scorias spongiosa* is the only edible fungus in the Capnodiaceae family and is widespread in many countries and regions (Batool et al., 2015; Yang, 2014). *S. spongiosa* was identified for the first time as a new species by pure culture and ITS sequence identification in 2011 (Zhong et al., 2020), and it is taxonomically affiliated with the genus Scorias Fr. (1825) of the Capnodiaceae family (Zhong et al., 2020). As a pathogen that affects bamboo, the growth of *S. spongiosa* depends on the honeydew secreted by *Pseudoregma bambucicola*, a pest that negatively impacts the healthy growth of bamboo plants (Harunobu, 1999). Although this fungus does not penetrate into and infect plant tissues, as it grows and ages, it forms black mycelia and thick fruiting bodies that cover plant tissues, thus hindering photosynthesis and, in severe cases, even leading to the death of the plant (Chomnunti et al., 2014; Zhang et al., 2021); thus, harvesting *S. spongiosa* fruiting bodies is ecologically beneficial. Given that *S. spongiosa* is rich in nutrients, containing high amounts of polysaccharides, amino acids, trace elements, and vitamins (Zhong et al., 2020; Sun et al., 2019; Wu et al., 2018; Huang et al., 2016), it has considerable developmental prospects. To date, *S. spongiosa* has not been artificially cultivated on a large scale, and all *S. spongiosa* products on the market are obtained from the wild (Huang et al., 2016). In our previous research, we found that the shape and color of *S. spongiosa* fruiting bodies differ at different growth stages. However, it is not known whether the nutritional components of *S. spongiosa* fruiting bodies also differ at different growth stages. A thorough and dynamic assessment of metabolites in *S. spongiosa* fruiting bodies has not yet been carried out.

The two main current branches of metabonomics studies are nontargeted and targeted metabonomics. Nontargeted metabonomics is an analytical strategy for identifying metabolites in as many samples as possible, which can guide the resolution of biological problems at the metabolic level, generally without bias (Zhao et al., 2019). In the field of edible mushrooms, a previous study characterized the metabolomic profiles of *Dictyophora rubrovolvata* under different drying treatments via nontargeted metabonomics and revealed the metabonomic profiles of *D. rubrovolvata* and potential biomarkers under different drying techniques (Dong et al., 2022). Related studies using nontargeted metabonomics have revealed the significantly different metabolic profiles of five commercial truffle species (Li et al., 2019). By combining nontargeted and targeted metabolomics, Carvalho et al. (2014) identified metabolites/metabolite patterns with potential species identification of 22 mushroom species.

In this study, nontargeted metabonomic analysis was conducted on *S. spongiosa* fruiting bodies at five growth stages, and the differences in metabolites and the related metabolic pathways during growth and development were analyzed. The main objective of this study was to provide metabonomic information to help determine the optimal harvesting period of *S. spongiosa* fruiting bodies for food processing, which may improve cultivation practices, enhance the quality of *S. spongiosa* fruiting bodies, and provide a theoretical basis for their development and utilization.

## 2 Materials and methods

### 2.1 Materials

*Scorias spongiosa* was planted at the *S. spongiosa* experimental base in Shaping town, Yibin city, Sichuan Province, China. The growth cycle of *S. spongiosa* is influenced by the growth environment (temperature, rainfall). The growth cycle of the fruiting bodies of *S. spongiosa* from October–November is 30–35 days (as observed during the experiment), and the primary growth stage of *S. spongiosa* is 5–7 days after fruiting bodies begin to form. Six clusters of *S. spongiosa* at the primary growth stage were handpicked on October 11, 2019. *S. spongiosa* at the subsequent growth stages were then collected every week. The five different growth stages of *S. spongiosa* were differentiated on the basis of the fruiting body color: pale yellow, yellow, deep yellow, yellowish-black and black. All collected samples were immediately stored in liquid nitrogen and then transferred to a refrigerator at −80°C.

### 2.2 Extraction and fractionation

The *S. spongiosa* samples stored in a freezer at −80°C were transferred to another freezer at −20°C and then transferred to a refrigerator at 4°C for gradual thawing. A total of 200 mg of each sample was accurately weighed, placed in a 2 mL EP tube, combined with 0.6 mL of methanol, and vortexed for 30 s. Glass beads were then added to the sample, which was then placed in a TisueLysis II tissue grinder, ground for 60 s at 25 Hz, ultrasonically crushed for 15 min at room temperature, and centrifuged at 25°C for 10 min. A total of 300 μL of the supernatant was filtered through a 0.22 μm membrane. A total of 20 μL of the filtrate of each sample to be tested was mixed into QC samples, and the remaining samples were analyzed via LC–MS (De Vos et al., 2007; Sangster et al., 2006).

### 2.3 Analytical procedure and MS conditions

Chromatographic conditions: 2 μL aliquots were injected onto a Waters ACQUITY UPLC HSS T3 column (150 mm × 2.1 mm, 1.8 μm) at 40°C and resolved via a gradient elution program with a flow rate of 0.25 mL/min. The temperature of the automatic sampler was set at 8°C, and the column temperature was set at 40°C. The mobile phases in negative ionization mode were 5 nM ammonium formate water (solvent A1) and acetonitrile (solvent B1). The mobile phases in positive ionization mode were a mixture of water/formic acid (99.9:0.1, v/v; solvent A2) and acetonitrile/formic acid (99.9:0.1, v/v; solvent B2). The gradient elution procedure was as follows: 0–1 min, 2% B1/B2; 1–9 min, 2–50% B1/B2; 9–12 min, 50–98% B1/B2; 12–13.5 min, 98% B1/B2; 13.5–14 min, 98–2% B1/B2; and 14–20 min, 2% B2- positive mode (14–17 min, 2% B1–negative mode). The positive ionization mode parameters were as follows: capillary temperature, 325°C; nebulizer voltage, 3,200 V; sheath gas, 30 arb; and auxiliary gas, 10 arb. The negative ionization mode parameters were as follows: capillary temperature, 325°C; nebulizer voltage, 2,500 V; sheath gas, 30 arb; and auxiliary gas, 10 arb (Want et al., 2010).

### 2.4 Data analysis

The original data were converted into mzxml format via ProteoWizard software, and peak identification, filtering, and alignment were conducted via the xcms package of R; a data matrix, including information such as the mass-to-core ratio, retention time, and peak area, was obtained (Thévenot et al., 2015). A total of 22,464 precursor molecules were obtained in positive ionization mode, and 18,652 precursor molecules were obtained in negative ionization mode. The data were exported to EXCEL for subsequent analysis. The peak areas of the data were normalized in batches to compare data of different orders of magnitude. Principal component analysis (PCA) and partial least squares discriminant analysis (PLS-DA) were conducted via SIMCA-P 14.1 software. Orthogonal partial least-squares discriminant analysis (OPLS-DA) in R was used for multivariate statistical analysis. Metabolites were first identified on the basis of accurate molecular weights (molecular weight errors of less than 20 ppm) and confirmed and annotated via databases such as HMDB,^1^ METLIN,^2^ and Mona^3^ in accordance with the MS/MS fragmentation patterns.

## 3 Results

### 3.1 Multivariate analysis at the five different growth stages

**Figure 1:**
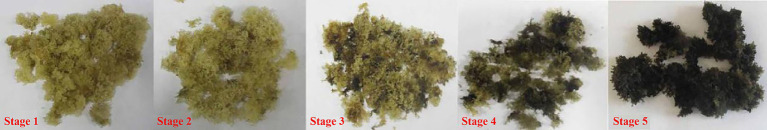
Different developmental stages of *S. spongiosa.* Stages 1–5 are pictures of the substrates on Days 5, 12, 19, 26, and 33 after substrate formation, respectively.

Supplementary Figure 1 presents the total base peak chromatograms (BPCs) in positive and negative ionization modes for the five different growth stages. A total of 15,397 and 11,589 metabolite ion features were identified in *S. spongiosa* in the positive and negative ionization modes, respectively (Figure 1).

### 3.2 Unsupervised statistical analysis

**Figure 2:**
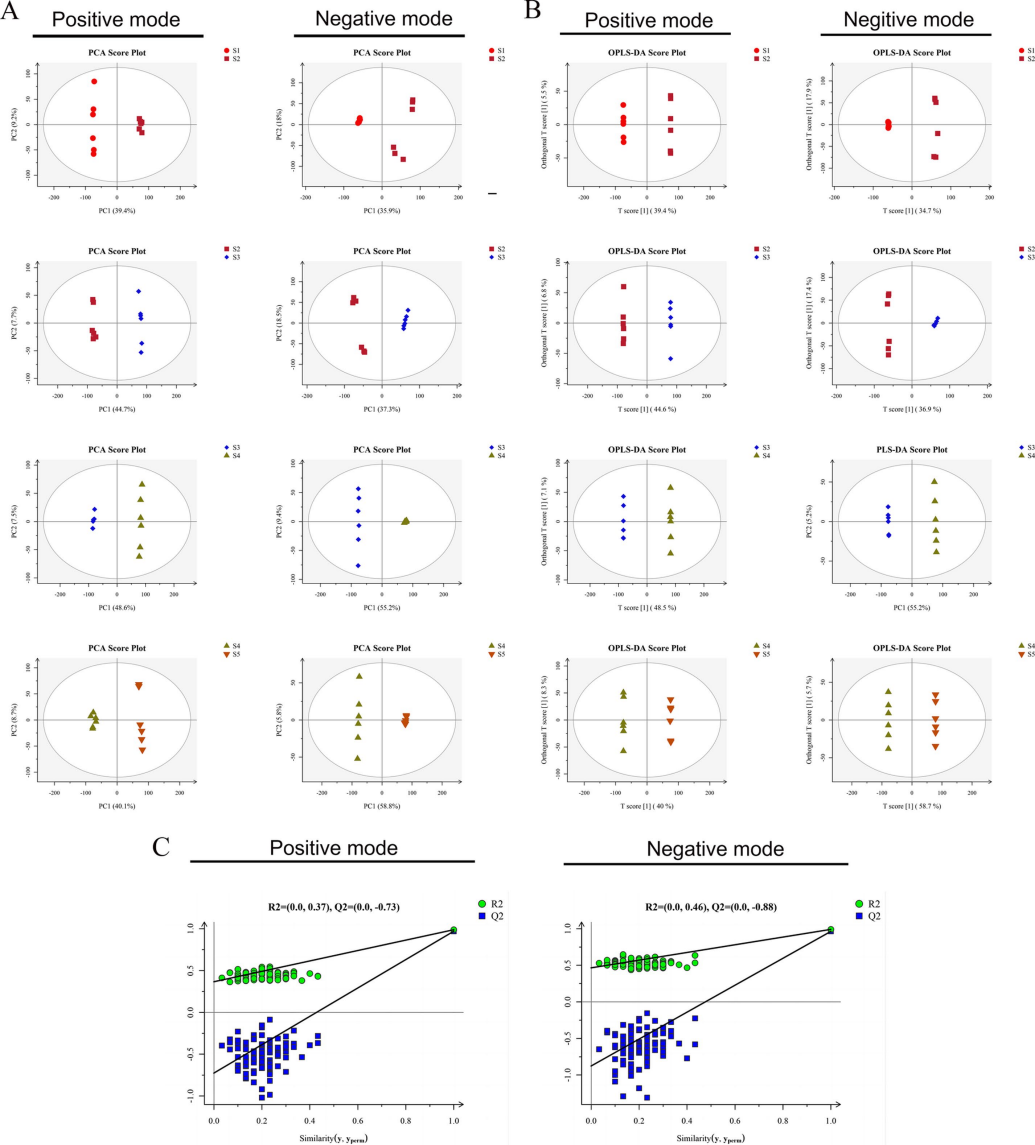
Data quality analysis. **(A)** PCA score plots of the data obtained in positive and negative ionization modes. **(B)** OPLS-DA models of the data obtained in positive and negative ionization modes for samples: score scatter plots. **(C)** OPLS-DA models of the data obtained in positive and negative ionization modes for samples: permutation tests.

The metabolite profiles acquired under both ionization modes were analyzed through PCA. The QC samples were in the middle of the five groups and clustered closely, indicating that the experiment was stable and reproducible (Supplementary Figure 2). The score plot in Figure 2A shows that the growth stages (S1–S2, S2–S3, S3–S4, and S4–S5) were clearly separated, suggesting apparent differences in the structures and compositions of metabolites among the different growth stages. The OPLS-DA results revealed that the samples at the different growth stages were separated in both ionization modes and were within the 95% confidence interval (Figure 2B). All of the OPLS-DA models were validated by response permutation testing, which revealed the absence of overfitting (Figure 2C) and false-positives in the experimental data. The model can thus be used to distinguish the five different growth stages of *S. spongiosa* (Boulesteix and Strimmer, 2007).

### 3.3 Differentially abundant metabolite analysis

**Figure 3:**
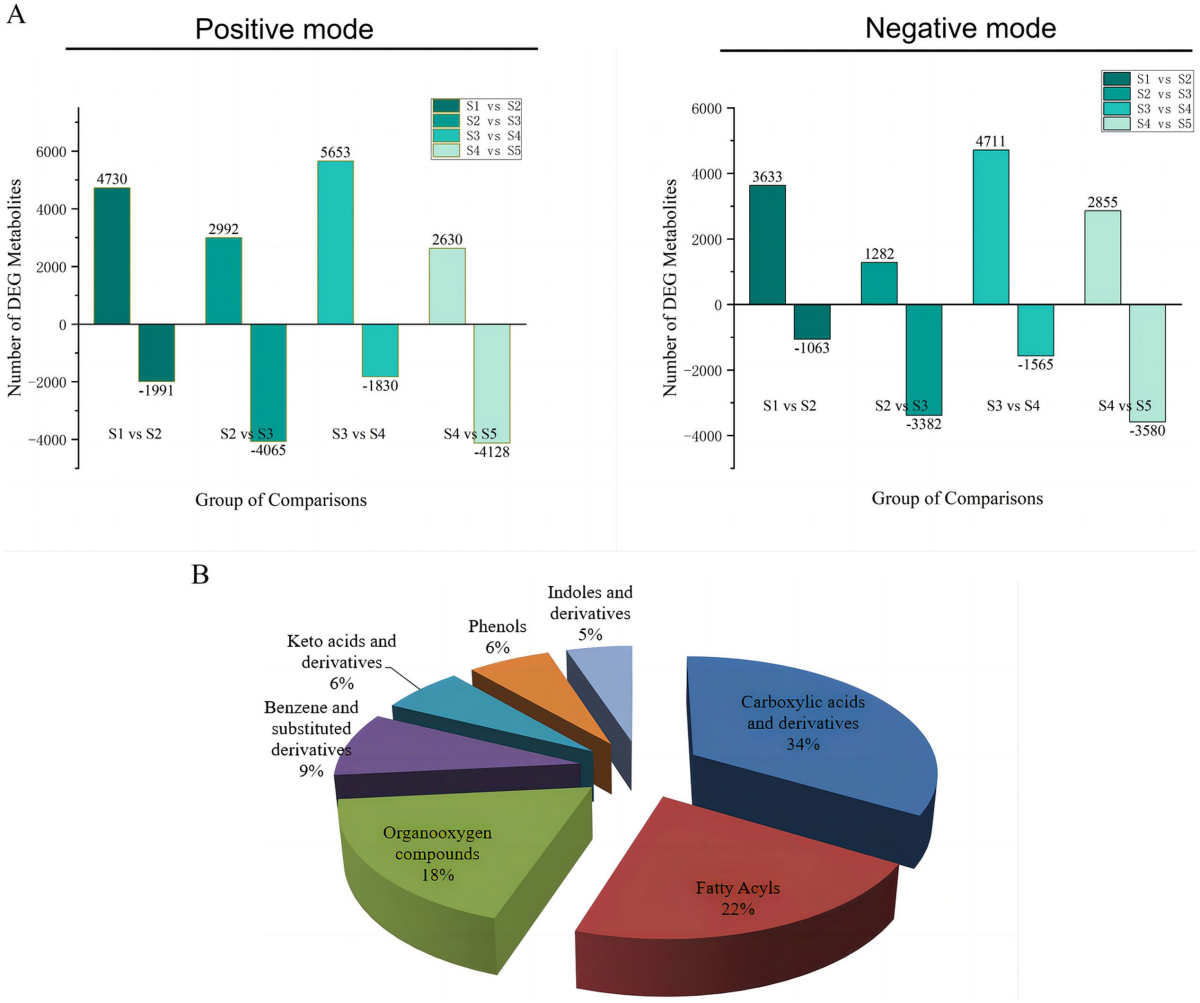
**(A)** Quantitative statistics of the different metabolites in positive and negative ionization modes. **(B)** Category statistics of differentially abundant metabolites.

**Figure 4:**
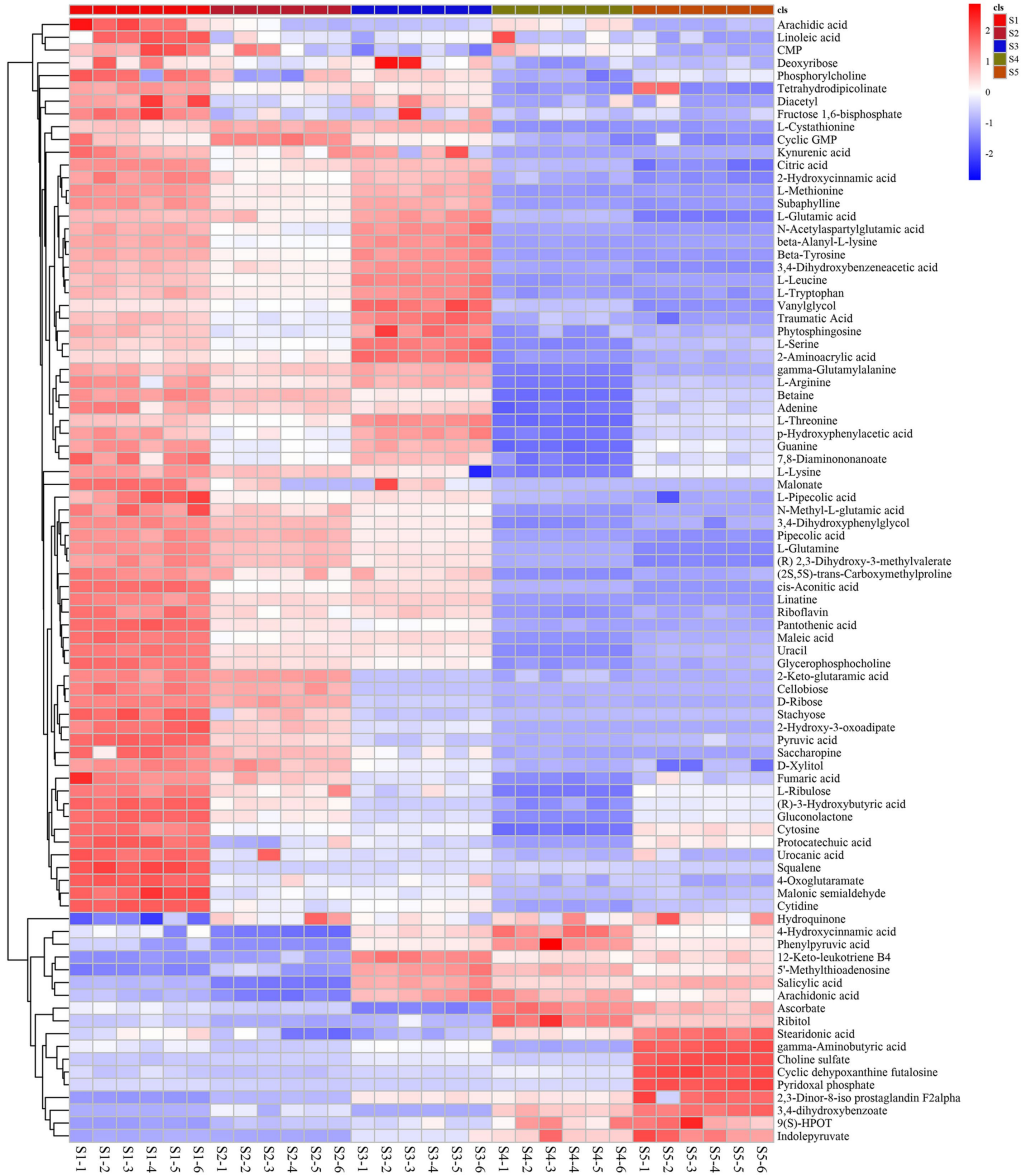
Heatmap of the differentially abundant metabolites in the different growth stages of *Scorias spongiosa*. Heatmap of the significantly differentially abundant metabolites at the five developmental stages of *S. spongiosa* identified in the LC/MS analysis. Each column represents the developmental stage, and the fold change in the average peak area is denoted by the number and color of the heat scale. The deeper the red color is, the higher the level of that metabolite in the developing fruits. Similarly, the deeper the blue color is, the lower the level of that metabolite in the developing fruits.

The differentially abundant metabolites at the different growth stages of *S. spongiosa* are shown in Figure 3A. The differentially abundant metabolites were assigned to various chemical categories, including carboxylic acids and derivatives (34%), fatty acyls (22%), organo-oxygen compounds (18%), benzene and substituted derivatives (9%), keto acids and derivatives (6%), phenols (6%), indoles and derivatives (5%) (Figure 3B). Subsequent screening for metabolites with significant differences between different stages revealed a total of 88 significantly differentially abundant metabolites among the five developmental stages; 137, 138, 161, and 142 metabolites were significantly differentially expressed in the S1–S2, S2–S3, S3–S4, and S4–S5 comparisons, respectively (Supplementary File). Hierarchical heatmap clustering analysis revealed significant differences in metabolite abundance across the five developmental stages of *S. spongiosa*, and all the biological replicates were grouped together, indicating the good quality and high reliability of the metabolome data (Figure 4).

### 3.4 KEGG enrichment analysis

**Figure 5:**
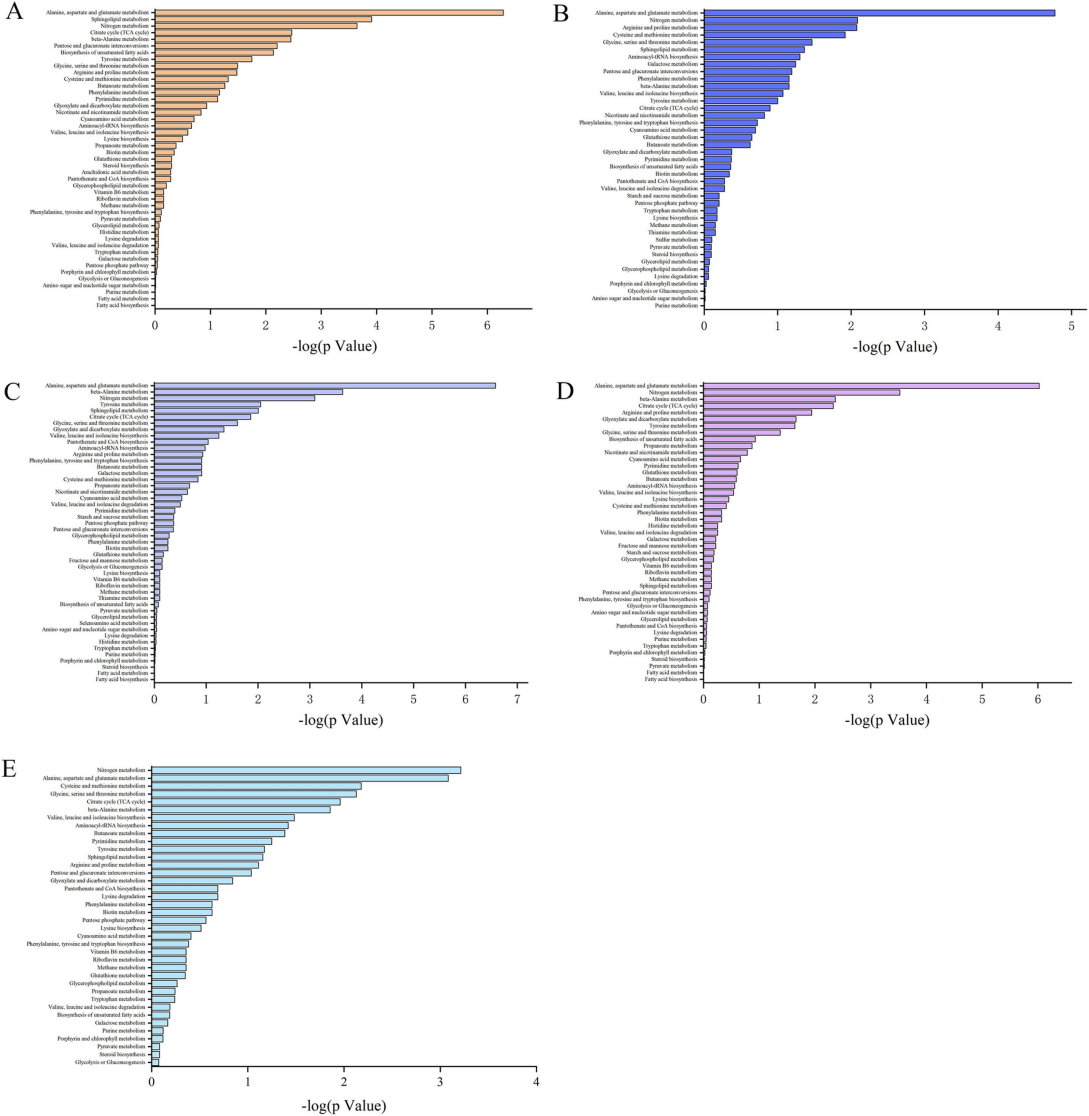
Distribution of the differentially abundant metabolites in various metabolic pathways. **(A–E)** Present the distributions of the differentially expressed metabolites in the S1–S2, S2–S3, S3–S4, S4–S5, and S1–S5 comparisons, respectively.

KEGG pathway enrichment analysis was used to illustrate the biological functions of the proteins that were differentially expressed at the different growth stages. The metabolites that were differentially expressed in the S1–S2, S2–S3, S3–S4, and S4–S5 comparisons were enriched in 45, 41, 48, and 45 metabolic pathways, respectively. The differentially abundant metabolites were enriched in 38 metabolic pathways during the entire growth period of *S. spongiosa* (Figure 5). Among all the enriched pathways, 15 metabolic pathways were common to all the analysis groups (Supplementary Table 2).

### 3.5 Distribution of bioactive compounds in *Scorias spongiosa* fruiting bodies

**Figure 6:**
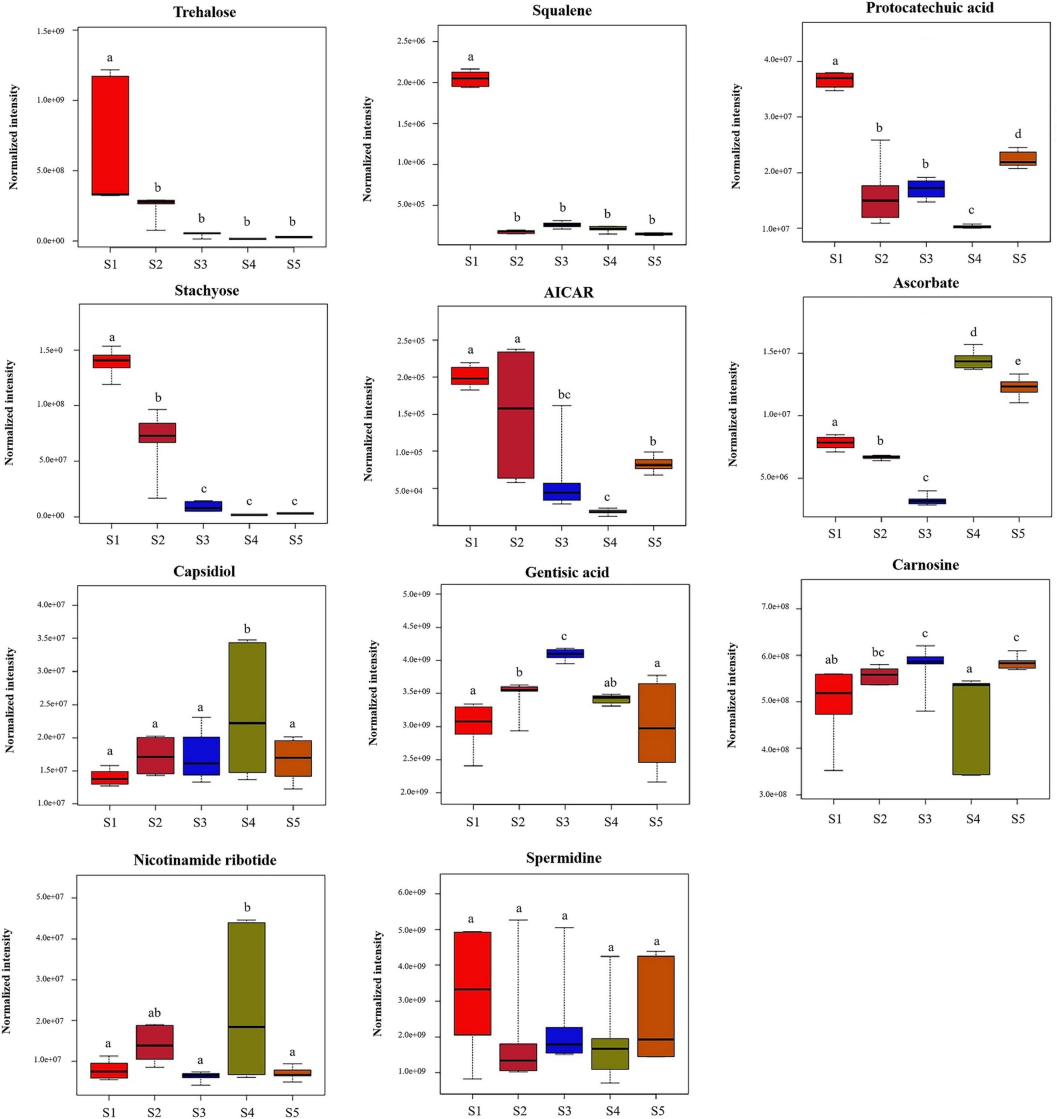
Differences in the contents of bioactive metabolites at the five different developmental stages. Different superscripts (a, b, c, d, e) indicate a significant difference (*p* < 0.05). The same superscript indicates no significant difference (*p* > 0.05).

Eleven compounds with biological activities were identified from among the significantly differentially abundant compounds. The contents of these eleven active substances during the five growth stages are shown in Figure 6. The levels of trehalose, spermidine, squalene, AICAR, protocatechuic acid, and stachyose were high at the S1 stage, of which the levels of squalene, protocatechuic acid, and stachyose were significantly higher than those at the other stages (*p* < 0.05). These six bioactive compounds tended to decrease after the S1 stage. Moreover, the contents of ascorbate at S3 and S4 were significantly higher than those at the other stages (*p* < 0.05). The carnosine content did not significantly differ among the five growth stages (*p* > 0.05). The results showed that there are differences in the bioactive substances contained in *S. spongiosa* fruiting bodies at different stages; therefore, the efficacy of different stages may differ.

### 3.6 Carbohydrate metabolism-related metabolites in *Scorias spongiosa* fruiting bodies

**Figure 7:**
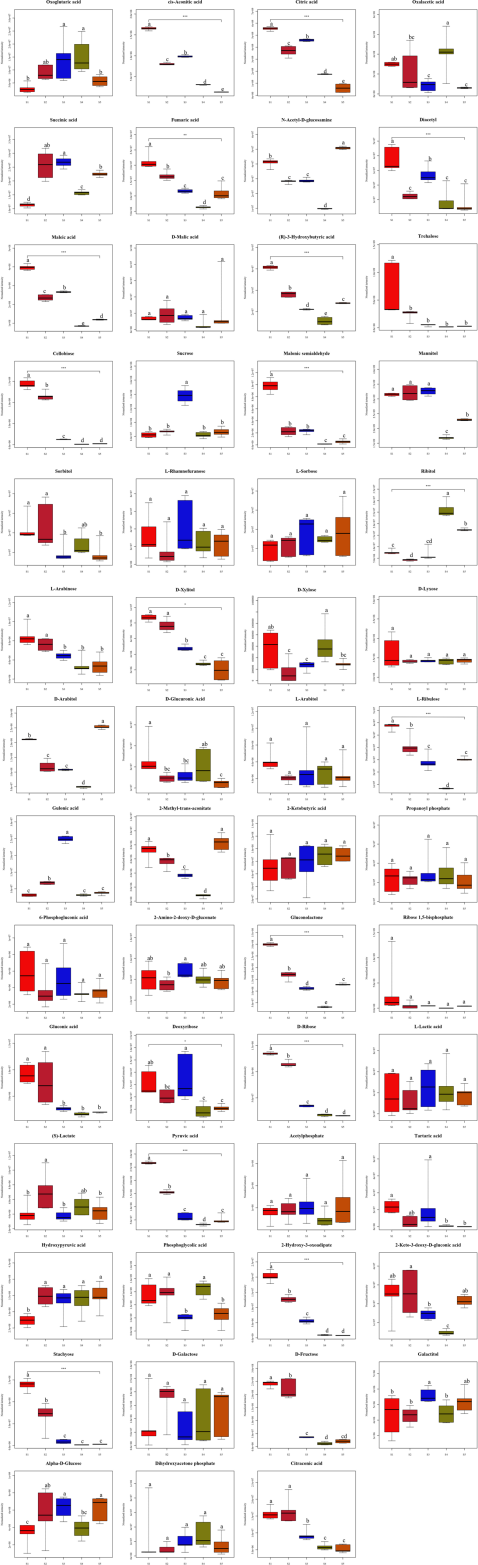
Differences in the contents of carbohydrate metabolites at the five different developmental stages. Different superscripts (a, b, c, d, e) indicate a significant difference (*p* < 0.05). The same superscript indicates no significant difference (*p* > 0.05).

Sugar metabolism provides an essential source of energy and metabolites for fruiting body induction, development and maturation (Sakamota, 2018). Therefore, in the present study, by comparing the five growth stages of *S. spongiosa*, 55 metabolites related to sugar metabolism were identified, among which 44 compounds were significantly differentially abundant and 11 substances were not significantly differentially abundant (Figure 7). Among the 55 metabolites related to sugar metabolism, ten, seven, and six were enriched in pentose and gluconate interconversions, the pentose phosphate pathway, and the TCA cycle, respectively (Supplementary File 1 and Figure 8).

## 4 Discussion

The edible fungus *S. spongiosa* has attracted attention in recent years because of its unique flavor and high nutritional value; however, related research is still in its infancy. Therefore, it is important to collect metabolic data to further explore the nutritional value, biological activity and potential medicinal value of *S. spongiosa*. With the development of histological technology, liquid chromatography–mass spectrometry (LC–MS) has been widely used in metabolomics research. A previous study analyzed the metabolic profiles of five commercial truffle species via nontargeted metabolomics and reported that *Tuber melanosporum* contains more compounds at higher concentrations, suggesting that *T. melanosporum* has a greater value for utilization as a drug or a foodstuff. In this study, we analyzed the dynamics of metabolites in *S. spongiosa* substrates at five growth stages via LC/MS-based nontargeted metabolomics, and the results revealed that the five growth stages of *S. spongiosa* shared 15 enriched pathways. These 15 metabolic pathways are speculated to play important roles in the growth of *S. spongiosa*. The growth of *S. spongiosa* can be regulated by modulating these metabolic pathways. This finding is highly important for further studies on the growth regulatory mechanisms of *S. spongiosa*.

By analyzing the bioactive components and sugar-related metabolism, we attempted to explain the antitumour, anti-inflammatory, bacteriostatic and antioxidant effects of *S. spongiosa* from a metabolic point of view, as well as to determine the ideal harvest time for the desired *S. spongiosa* fruiting bodies. In this study, 11 bioactive compounds were identified from among the differentially abundant compounds (Table 1). Trehalose can protect motor neurons and improve neuroinflammation (Liu et al., 2019; Li et al., 2020). Squalene is a terpenoid with antioxidant, anticancer and antifatigue activities (Ramli et al., 2018; Sakul et al., 2019). Protocatechuic acid, a phenolic acid, has antioxidant and antiaging effects (Girsang et al., 2020; Sinha et al., 2020; Al Olayan et al., 2020). Stachyose has antidiabetic and immunomodulatory effects (Liang et al., 2019; Shang et al., 2020). Gentisic acid, a phenolic acid, has numerous functions, such as antioxidation, anti-inflammatory, and bacteriostatic effects (Abedi et al., 2020; Cavalcante et al., 2018; Kabra et al., 2014).Ascorbate, a polyhydroxy compound, has antioxidation and immunity-enhancing functions (Thomas et al., 2020; Escobar and Paltas, 2019). Capsidiol has anti-inflammatory, anticancer and antifatigue effects (Yang and Song, 2020). In the present study, the levels of six bioactive compounds (trehalose, spermidine, squalene, AICAR, protocatechuic acid, and stachyose) were high at the S1 stage, of which the levels of squalene, protocatechuic acid, and stachyose were significantly higher than those at the other stages. These six bioactive compounds tended to decrease after the S1 stage. Therefore, *S. spongiosa* can be harvested during the S1 period to retain most of the bioactive substances, especially for the development of nutraceuticals because of its antioxidant, antidiabetic, and immunoregulatory properties. Moreover, the contents of ascorbate at S3 and S4 were significantly higher than those at the other stages, so *S. spongiosa* can be harvested at this stage to develop antioxidant and immune-boosting nutraceuticals.

**Table 1:** Specific nutritional components and functions of *S. spongiosa*.

Chemical compound	Function
Trehalose	Protecting motor neurons, Anti-inflammatory (Sinha et al., 2020; Li et al., 2020)
Carnosine	Antioxidant, Anti-brain aging, Protecting nerves (Tanaka and Kawahara, 2019; Kim and Kim, 2020; Scuto et al., 2020)
Gentisic acid	Antioxidant, Anti-inflammatory, Antibacterial, Protecting nerves (Abedi et al., 2020; Cavalcante et al., 2018; Kabra et al., 2014)
Spermidine	Antiageing (Thomas et al., 2020; Metur and Klionsky, 2020)
Nicotinamide ribotide	Antiageing (Tamas et al., 2020)
Ascorbate	Antioxidant, Immune-boosting, Relieve muscle fatigue (Hureau et al., 2020; Escobar and Paltas, 2019)
Capsidiol	Anti-inflammatory, Anticancer, Resist fatigue (Yang and Song, 2020)
Squalene	Antioxidant, Anti-cancer, Resist fatigue (Ramli et al., 2018; Sakul et al., 2019)
AICAR	Antiageing, Anti-inflammatory (Mohammadhossein et al., 2020; Martin et al., 2019)
Protocatechuic acid	Antioxidant, Antiageing, Antidiabetic, Anti-inflammatory, Antiapoptotic (Al Olayan et al., 2020; Girsang et al., 2020; Seoungwoo et al., 2020)
Stachyose	Antidiabetic, Immunoregulation (Liang et al., 2019; Shang et al., 2020)

Sugars serve as important carbon sources for fruiting body development, and they may be used as energy donors or raw materials for polysaccharide synthesis to promote the rapid growth of fruiting bodies (Sakamota, 2018). *S. spongiosa* is rich in polysaccharides (Zhong et al., 2020). Xu et al. (2022) treated C57BL/6 J mice with low-dose and high-dose *S. spongiosa* polysaccharides and reported that these polysaccharides had no effect on the growth performance of the mice regardless of dose, and that their antioxidant and anti-inflammatory activities were elevated. In subsequent studies, *S. spongiosa* polysaccharides were shown to have protective effects against colitis in mice by reshaping the intestinal microbiome and maintaining the balance of intestinal barrier integrity (Xu et al., 2023). In this study, the results revealed that the metabolites related to sugar metabolism are involved in three main pathways: pentose and gluconate interconversions, the pentose phosphate pathway, and the TCA cycle. Polysaccharides, pentoses and glucose are all saccharides that can be interconverted via different carbohydrate metabolic pathways, although these pathways are linked via common intermediates (such as glucose 6-phosphate and glyceraldehyde 3-phosphate, etc.) (Chandel et al., 2021). In particular, the pentose phosphate pathway linked pentose to hexose, and the interconversions of different hexoses with glucose mediate the metabolism of hexoses, and the activated glucose can be further synthesized and become polysaccharides (Wang et al., 2017; Stincone et al., 2015). These linkages allow *S. spongiosa* to efficiently use and convert sugars to meet its energy and biosynthetic needs. Therefore, these three pathways were speculated to be related to the change in polysaccharide content during the growth of *S. spongiosa* in the present study. The TCA cycle provides energy for biological activities and is the most effective way for the body to obtain energy through the use of sugar (Avila et al., 2017). With the growth of *S. spongiosa*, the contents of oxoglutarate, cis-aconitic acid, and citric acid involved in the TCA cycle all decreased, indicating that the energy supply decreased gradually at the later stages of growth. For this reason, *S. spongiosa* can be harvested at the early growth stage to retain a higher saccharides content, thus ensuring its nutritional value.

Moreover, we found that *S. spongiosa* fruiting bodies presented different colors at different stages of development, with the color gradually changing to black as the fruiting bodies matured. Previous studies showed that the production of melanin is linked to the metabolism of amino acids, such as tyrosine, phenylalanine, and tryptophan. For example, under the catalytic reaction of tyrosinase, the tyrosine can synthesize melanin through a series of complex biological processes, in contrast, the phenylalanine can inhibits the tyrosinase activity, thereby inhibiting the melanin formation (Faure et al., 2024; Rzepka et al., 2016; Farishian et al., 1980). Moreover, tryptophan is also involved in the synthesis of melatonin (Murch et al., 2000), and L-tryptophan is proven to be a precursor for the synthesis of melatonin, as described by the physician Lerner et al. (1958). In this study, our differentially abundant metabolite analysis revealed that tyrosine was distributed across all five stages of *S. spongiosa*, with a high level in S1, S2, or S3 stages (1.21 ~ 1.71 × 10^8^) and a significant decreased level in S4 and S5 stages (6.60 ~ 6.87 × 10^7^), a similar decrease was also observed in phenylalanine. In addition, the level of tryptophan was high in each of the first three stages (S1, S2, and S3), but such level significantly dropped in stage S4 and even disappeared in stage S5 (Supplementary File 2). Therefore, we speculated that as the fruiting bodies grow, the levels of phenylalanine decreased, which recovered the activity if the tyrosinase and catalyzed a large amount of tyrosine into melanin; conversely, tryptophan greatly reduced or even disappeared in stages S4 and S5, resulting in a significant reduction of melatonin, which ultimately reduced a black color in the later stages of the fruiting bodies of *S. spongiosa* (S4 and S5). The yellow color of the fruiting bodies in stages S1, S2, S3 may be due to the relatively high levels of tyrosine and tryptophan.

## 5 Conclusion

Nontargeted metabonomics analysis revealed that the five growth stages of *S. spongiosa* shared 15 enriched pathways. These 15 metabolic pathways are speculated to play important roles in the growth of *S. spongiosa*. Eleven bioactive substances were identified from among the differentially expressed compounds. The content of bioactive substances differed during the different growth stages. *S. spongiosa* can be harvested during the S1 period to retain most of the bioactive substances. The results of the analysis revealed that the metabolites related to sugar metabolism are involved in three main pathways: pentose and gluconate interconversions, the pentose phosphate pathway, and the TCA cycle. These three pathways are speculated to be related to the change in polysaccharide content during the growth of *S. spongiosa*.

## Data Availability

The raw data supporting the conclusions of this article will be made available by the authors, without undue reservation.

## Footnotes

^1^http://www.hmdb.ca

^2^https://metlin.scripps.edu/landing_page.php?pgcontent=mainPage

^3^https://mona.fiehnlab.ucdavis.edu
