# Supplementary material for: Nontargeted metabonomics analysis of Scorias spongiosa fruiting bodies at different growth stages
Source: Front Microbiol. 2024 Oct 30;15:1478887. doi: 10.3389/fmicb.2024.1478887 (PMC11557477; doi:10.3389/fmicb.2024.1478887)
Supplement: Supplementary file 1 [file Data_Sheet_1.PDF]

Different metabolites involved in Carbohydrate metabolism in the growth stage of *Scorias spongiosa*

Name	S1 V S2	S2 V S3	S3 V S4	S4 V S5	S1V S2 VS3 VS4 VS5	Sub-pathway
Oxoglutaric acid	√			√		Citrate cycle (TCA cycle)
cis-Aconitic acid	√	√	√	√	√	Citrate cycle (TCA cycle)
Citric acid	√	√	√	√	√	Citrate cycle (TCA cycle)
Oxalacetic acid			√	√		Citrate cycle (TCA cycle)
Succinic acid	√	√	√	√	√	Citrate cycle (TCA cycle)
Fumaric acid	√	√	√	√	√	Citrate cycle (TCA cycle)
N-Acetyl-D-glucosamine	√		√	√		Amino sugar and nucleotide sugar metabolism
Diacetyl	√	√	√		√	
Maleic acid	√	√	√		√	Butanoate metabolism
D-Malic acid						Butanoate metabolism
(R)-3-Hydroxy butyric acid	√	√	√	√	√	Butanoate metabolism
Trehalose		√	√	√		Starch and sucrose metabolism
Cellobiose	√	√	√	√	√	Starch and sucrose metabolism
Sucrose		√	√			Starch and sucrose metabolism
Malonic semialdehyde	√		√	√	√	Inositol phosphate metabolism
Mannitol			√	√		Fructose and mannose metabolism
Sorbitol						Fructose and mannose metabolism
L-Rhamnofuranose						Fructose and mannose metabolism
L-Sorbose						Fructose and mannose metabolism

Name	S1 V S2	S2 V S3	S3 V S4	S4 V S5	S1V S2 VS3 VS4 VS5	Sub-pathway
Ribitol	√		√	√	√	Pentose and glucuronate interconversions
L-Arabinose		√				Pentose and glucuronate interconversions
D-Xylitol	√	√	√		√	Pentose and glucuronate interconversions
D-Xylose	√					Pentose and glucuronate interconversions
D-Lyxose						Pentose and glucuronate interconversions
D-Arabitol	√		√	√		Pentose and glucuronate interconversions
D-Glucuronic Acid	√					Pentose and glucuronate interconversions
L-Arabitol	√					Pentose and glucuronate interconversions
L-Ribulose	√	√	√	√	√	Pentose and glucuronate interconversions
Gulonic acid	√	√	√	√		Pentose and glucuronate interconversions
2-Methyl-trans-aconitate	√	√	√	√		Propanoate metabolism
2-Ketobutyric acid						Propanoate metabolism
Propanoyl phosphate						Propanoate metabolism
6-Phosphogluconic acid						Pentose phosphate pathway
2-Amino-2-deoxy-D-gluconate		√				Pentose phosphate pathway
Gluconolactone	√	√	√	√	√	Pentose phosphate pathway
Ribose 1,5-bisphosphate			√	√		Pentose phosphate pathway
Gluconic acid		√	√			Pentose phosphate pathway

Name	S1 V S2	S2 V S3	S3 V S4	S4 V S5	S1V S2 VS3 VS4 VS5	Sub-pathway
Deoxyribose			√		√	Pentose phosphate pathway
D-Ribose	√	√	√		√	Pentose phosphate pathway
L-Lactic acid						Pyruvate metabolism
(S)-Lactate	√	√				Pyruvate metabolism
Pyruvic acid	√	√	√		√	Pyruvate metabolism
Acetylphosphate						Pyruvate metabolism
Tartaric acid	√					Glyoxylate and dicarboxylate metabolism
Hydroxypyruvic acid	√					Glyoxylate and dicarboxylate metabolism
Phosphoglycolic acid		√	√	√		Glyoxylate and dicarboxylate metabolism
2-Hydroxy-3-oxoadipate	√	√	√		√	Glyoxylate and dicarboxylate metabolism
2-Keto-3-deoxy-D-gluconic acid		√	√	√		Galactose metabolism
Stachyose	√	√	√	√	√	Galactose metabolism
D-Galactose		√				Galactose metabolism
D-Fructose		√	√			Galactose metabolism
Galactitol		√	√			Galactose metabolism
Alpha-D-Glucose			√	√		Glycolysis / Gluconeogenesis
Dihydroxyacetone phosphate						Glycolysis / Gluconeogenesis
Citraconic acid		√	√			C5-Branched dibasic acid metabolism

"√" indicates metabolites with significant differences at this stage.
