# Supplementary material for: Nontargeted metabonomics analysis of Scorias spongiosa fruiting bodies at different growth stages
Source: Front Microbiol. 2024 Oct 30;15:1478887. doi: 10.3389/fmicb.2024.1478887 (PMC11557477; doi:10.3389/fmicb.2024.1478887)
Supplement: Supplementary file 5 [file Table_2.DOCX]

Supplementary Table 1. Significant differential metabolites at each growth stage

| Group | Difference number |
| --- | --- |
| S1 vs S2 | 137 |
| S2 vs S3 | 138 |
| S3 vs S4 | 161 |
| S4 vs S5 | 142 |
| S1 vs S2 vs S3 vs S4 vs S5 | 88 |

Supplementary Table 2. Fifteen metabolic pathways shared by all analysis groups.

| Name | Name |
| --- | --- |
| Alanine, aspartate and glutamate metabolism | Biotin metabolism |
| Cysteine and methionine metabolism | Phenylalanine metabolism |
| Glycine, serine and threonine metabolism | Methane metabolism |
| Citrate cycle (TCA cycle) | Glutathione metabolism |
| Butanoate metabolism | Glycerophospholipid metabolism |
| Arginine and proline metabolism | Glycerolipid metabolism |
| Pentose and glucuronate interconversions | Nicotinate and nicotinamide metabolism |
| Glyoxylate and dicarboxylate metabolism |  |
